# Retinal thinning in phenylketonuria and Gaucher disease type 3

**DOI:** 10.1007/s00417-021-05424-5

**Published:** 2021-10-12

**Authors:** Susanne Hopf, Alexander K. Schuster, Julia B. Hennermann, Norbert Pfeiffer, Susanne Pitz

**Affiliations:** 1 Department of Ophthalmology, University Medical Center of the Johannes Gutenberg-University Mainz, Langenbeckstraße 1, 55131 Mainz, Germany; 2 Villa Metabolica, Department of Pediatric and Adolescent Medicine, University Medical Center Mainz, Mainz, Germany; 3 Orbital Center, Ophthalmic Clinic, Bürgerhospital Frankfurt, Frankfurt, Germany

**Keywords:** Ganglion cell layer, Ganglion cell complex, Retinal thinning, PKU, Gaucher, Lysosomal storage disease

## Abstract

**Purpose:**

Retinal alterations in inherited metabolic diseases associated with neurodegeneration are poorly studied. The objective was to study retinal thickness, specifically the components of the ganglion cell complex (GCC)—nerve fiber layer (NFL), ganglion cell layer (GCL), and inner plexiform layer (IPL)—using spectral-domain optical coherence tomography (SD-OCT) in two different diseases with potential dopaminergic depletion, phenylketonuria (PKU) and Gaucher disease type 3 (GD3).

**Methods:**

Retinal layers in 19 patients with PKU, 15 patients with GD3, and 93 healthy individuals were measured using peripapillary ring scan and macular SD-OCT. Linear mixed models were computed including an adjustment for age, sex, and spherical equivalent. We calculated Spearman’s rank correlations between retinal layer measurements and clinical and/or laboratory parameters.

**Results:**

Thinning of total retinal thickness was found in the macular inner ring (*p* = 0.002), and outer ring (*p* = 0.012), sparing the fovea (*p* = 0.12) in PKU, while in GD3, all subfields were thinned (fovea *p* < 0.001, inner ring *p* = 0.047, outer ring 0.07). In both conditions, thinning was most evident in the NFL, GCL, and IPL, while OPL (outer plexiform layer) was thickened. Peripapillary retinal nerve fiber layer measurements remained normal. GCL and IPL in PKU correlated with tyrosine serum concentration.

**Conclusion:**

Thinning of the NFL, GCL, and IPL, with thickened OPL, are both found in PKU and in GD3. Low dopamine concentrations in the retina might promote these effects. However, these data do not give evidence that retinal measurements can be used as a biomarker for disease severity in patients with GD3.

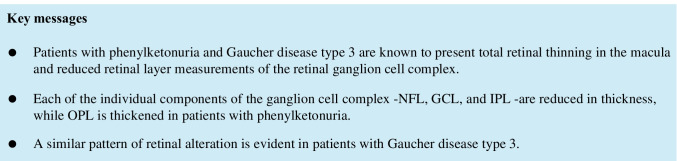

## Introduction

Optical coherence tomography (OCT), a non-invasive technique with a high resolution, enables to describe retinal structures almost at a cellular level in vivo. OCT layers correlate well with histological findings of the retina [1, 2]. The use of this imaging technique to demonstrate neurodegeneration of the retina or the optic disc in neurological and neuro-ophthalmological conditions is growing [3]. Retinal correlates have been proved primarily in Parkinson’s disease, Alzheimer’s disease, and multiple sclerosis, but also in rare conditions such as amyotrophic lateral sclerosis, and Huntington’s disease [3–5]. They resemble changes found in glaucoma by thinning of the macular ganglion cell complex [6], defined by the three innermost retinal layers (nerve fiber layer, NFL; ganglion cell layer, GCL; and inner plexiform layer, IPL) [7]. On the one hand, thinning of the combined ganglion cell-inner plexiform layer (GCIPL) and thinning of the peripapillary retinal nerve fiber layer (pRNFL) correlate with neurodegenerative processes of the entire CNS, such as in multiple sclerosis [3, 4], or with nigrostriatal dopaminergic degeneration related to Parkinson’s disease [8]. On the other hand, retinal layer measurements may provide structural evidence for dysfunction in the fovea and parafovea and retinal dopamine loss, as is assumed in Parkinson’s disease [8–10]. The retina is one of the tissues in the body with highest dopamine concentration [11]. Dopamine receptors are expressed in retinal cells throughout the retina with varying functions depending on the receptor subtype and the cell type [12]. The retinal dopaminergic system is involved in eye growth, light adaptation, circadian rhythmicity, and cell survival [13]. Dopamine has an important role in uncoupling horizontal and amacrine cell junctions [12]. Dopaminergic amacrine cells are located in the inner nuclear layer (INL) [14, 15].

Ganglion cell complex (GCC) thinning has recently been reported for the first time in the inherited metabolic diseases phenylketonuria[16] and Gaucher disease type 3 [17]. In both conditions, a dopaminergic depletion can be speculated. In PKU, dopamine depletion has been related to reduced tyrosine uptake into the brain and reduced tyrosine-hydroxylase activity [18]. In Gaucher type 1, GCC thinning was demonstrated primarily in the presence of parkinsonian features or other clinical markers of early neurodegeneration (hyposmia, cognitive impairment, parkinsonian motor signs) [17, 19, 20]. Thus, in both diseases, changes in neurotransmission may be one mechanism leading to functional and morphological retina alteration.

Our aim was to study segmental retinal layers, specifically the components of the ganglion cell complex (GCC)—nerve fiber layer (NFL), ganglion cell layer (GCL), and inner plexiform layer (IPL)—by means of spectral-domain (SD) optical coherence tomography (OCT) in two different diseases with potential dopaminergic depletion, phenylketonuria (PKU) and Gaucher disease type 3 (GD3). The second aim was to identify whether retinal layer measurements correlate with established disease features and if they may be used as a biomarker for disease severity. This should be of special interest for patients with GD3.

## Methods

### Study population

This study included 19 patients aged 6 to 46 years with mild, or classical, phenylketonuria or tetrahydrobiopterin-deficient hyperphenylalaninemia, 15 patients aged 6 to 44 years with Gaucher disease type 3, and 93 controls aged 6 to 75 years. The patients’ inclusion criteria were genetical and/or biochemical prove of their disease. Participants under 6 years of age were excluded. Controls were included if they had no ocular disease and age-appropriate visual function, as well as no relevant systemic disease (e.g., neurodegeneration). Controls were recruited stratifying for age (seven subgroups with at least 10 participants were built for an even age distribution).

The study was approved by the Medical Ethical Committee of the State Chamber of Medicine of Rhineland Palatinate in Mainz, Germany (reference number 837.373.14). All persons or their parents/guardians gave their written informed consent prior to inclusion in the study. The research adhered to the tenets of the Declaration of Helsinki.

### Ophthalmic examination procedure

The examination included non-cycloplegic auto-refraction measurements (NIDEK AR-360A, Nidek Co., Japan), best-corrected visual acuity testing, slit lamp biomicroscopy, and fundus examination, as well as orthoptic examination, which were published elsewhere [21–23]. Spherical equivalents defined as the sum of the spherical power and half of the cylindric power were used in the statistic models.

Imaging of the optic nerve head and the macula was carried out using spectral-domain (SD) optical coherence tomography (OCT) (Spectralis, Heidelberg Engineering GmbH, Heidelberg, Germany) with automatic real-time function for image averaging. We acquired a peripapillary OCT and a macular OCT. The peripapillary retinal nerve fiber layer (pRNFL) was imaged with a diameter of 12° (corresponding to 3.47 mm in the standard eye), and a standard corneal curvature of 7.7 mm. For the macular OCT, 49 horizontal single scans were acquired. After semi-automated segmentation of the retinal layers as provided by the OCT software (Heidelberg Eye Explorer version 1.10.2.0, viewing module 6.9.5.0; HEYEX, Heidelberg, Germany), all scans were assessed regarding their quality by a board-certified ophthalmologist (SH). Those with segmentation errors were corrected, or excluded in cases of poor image quality. In cases of poor data in only one sector, this sector was excluded prior to analysis; if more sectors were affected, then the complete OCT dataset of this eye was excluded.

Mean retinal thickness of 9 macular layers was used for the analysis: total retinal layer thickness of the macula, nerve fiber layer (NFL), ganglion cell layer (GCL), inner plexiform layer (IPL), inner nuclear layer (INL), outer plexiform layer (OPL), outer nuclear layer (ONL), outer retinal layer (ORL) (being limited by the external limiting membrane and Bruch’s membrane, this layer corresponds to the photoreceptors), and retinal pigment epithelium (RPE).

The 6-mm macular scan measurements were classified according to the ETDRS segments (“Early Treatment Diabetic Retinopathy Study” subfields). Central zone, inner ring, and outer ring with diameters of 1, 3, and 6 mm, respectively, were included in the analysis. The average of all points within the central zone (1 mm diameter) was defined as foveal thickness, the inner ring (1 to 3 mm) as parafoveal thickness, and the outer ring (3 to 6 mm) as perifoveal thickness.

### Clinical data

Typical variables described for disease stage in PKU and GD3 were determined and obtained from the patient’s records. For PKU, we used *first*, current phenylalanine serum concentrations of each individual (mean 693 µmol/l ± 384 µmol/l); *second*, current tyrosine serum concentrations of each individual (mean 105 µmol/l ± 60 µmol/l); *third*, disease treatment (16 early-treated vs. three late-treated individuals with PKU); and *fourth*, disease type (mild phenylketonuria with untreated blood phenylalanine concentrations of less than 1000–1200 µmol/l) [24], classical phenylketonuria, and tetrahydrobiopterin-deficient hyperphenylalaninemia). For GD3, we used *first*, the modified severity scoring tool (mSST), which is based on twelve domains including horizontal gaze palsy, cranial nerve palsy, seizures and age at first seizures, cognitive ability, ataxia, tremor, spasticity, rigidity, dysphagia, dysarthria, and spinal alignment [25]. *Second*, we considered the phenotype severity (mild, intermediate, severe), of which intermediate phenotype was associated with homozygous L444P mutation [22]. *Third/fourth*, we included horizontal/vertical peak velocity of reflexive saccades in GD3 (69°/s ± 58°/s and 192°/s ± 92°/s, respectively).

### Statistical analysis

Medians, interquartile ranges, minimums, and maximums were calculated for all continuous variables. For variables distributed normally, means and standard deviations were computed. For dichotomous variables, absolute and relative frequencies were computed.

To analyze the differences of retinal thickness with respect to PKU and GD3, we used linear mixed models to control for the inclusion of one and two eyes of a study participant (as random effect). A further adjustment for age, sex, and spherical equivalent was included in the statistical analysis. Full thickness measurements are more susceptible to these parameters, than single layer measurements, which is why we focused on single layer correlation analysis as follows. Spearman’s rank correlation was conducted to correlate thinned or thickened layers (e.g., GCL or OPL, respectively) with disease-specific variables of PKU (current phenylalanine and tyrosine serum concentration, early- vs. late-treated PKU, and disease type) and GD3 (mSST, phenotype severity, horizontal and vertical eye movements). The other retinal layers, which were not different to controls, or which were underrepresented in the specific subfield (fovea), were not further analyzed. Correlation coefficient rho of ≥ 0.5 was regarded a moderate correlation, correlation coefficient of ≥ 0.3 was considered a weak correlation, and < 0.3 was considered a no correlation. Statistical analysis was performed using R version 4.0.4. All *p*-values should be regarded as continuous parameters that reflect the level of evidence from our explorative analysis and are therefore reported exactly.

## Results

All patients examined were included in the study. From the 19 PKU patients (mean age 20 ± 12 years), 15 GD3 patients (mean age 20 ± 10 years), and 93 controls (mean age 32 ± 17 years), we excluded 6, 8, and 8 eyes respectively for the pRNFL analysis due to poor image quality, and 0, 2, and 6 eyes respectively for the macular OCT analysis.

### Macular full thickness and macular layers

#### Phenylketonuria

The OCT measurements indicate a significant thinner total retinal thickness in the inner and outer ring, but not in the fovea. This pattern affected the NFL, GCL, IPL, and ONL, while OPL was thickened. The differences were most evident in the NFL, GCL, and IPL (see Table 1) and more evident than in GD3. The remaining layers (INL, and ORL) did not differ from controls. RPE was thinned in the inner ring segment, but the difference was not significant after adjustment for sex, age, and spherical equivalent.

**Table 1:** Distribution of retinal layer measurements from SD-OCT in phenylketonuria, Gaucher disease type 3, and controls. A linear mixed model was used for statistical analysis. In the adjusted model, age, sex, and spherical equivalent were included

Macular OCT layers and ETDRS segments Mean (SD) [µm]	Control eyes (*n* = 180)	PKU eyes (*n* = 36)	GD3 eyes (*n* = 30)	*p*-value PKU vs. control crude model (adj. model)	*p*-value GD3 vs. control crude model (adj. model)
Total retinal thickness					
Fovea	278.3 (19.2)	271.0 (16.7)	260.2 (10.3)	0.12 (0.38)	< 0.001 (< 0.001)
Inner ring	343.8 (12.8)	331.9 (18.3)	336.1 (15.0)	0.002 (0.001)	0.047 (0.009)
Outer ring	306.9 (20.7)	295.1 (16.9)	298.1 (18.9)	0.012 (< 0.001)	0.07 (0.014)
1) NFL					
Fovea	12.7 (2.1)	11.7 (1.6)	11.8 (1.6)	0.05 (0.24)	0.13 (0.10)
Inner ring	21.7 (1.8)	20.2 (1.8)	21.3 (1.6)	< 0.001 (0.006)	0.33 (0.40)
Outer ring	36.3 (4.0)	33.0 (3.9)	34.3 (5.4)	0.004 (0.001)	0.08 (0.019)
2) GCL					
Fovea	16.1 (4.4)	14.1 (2.7)	13.9 (3.0)	0.07 (0.09)	0.06 (0.008)
Inner ring	52.3 (4.2)	46.5 (6.9)	50.0 (5.9)	< 0.0001 (< 0.001)	0.10 (0.004)
Outer ring	35.9 (3.1)	33.7 (3.6)	34.5 (4.0)	0.019 (0.001)	0.16 (0.014)
3) IPL					
Fovea	22.0 (3.7)	20.4 (2.3)	19.7 (2.2)	0.07 (0.14)	0.014 (0.002)
Inner ring	42.7 (2.8)	39.4 (3.3)	41.3 (3.5)	< 0.0001 (< 0.001)	0.12 (0.013)
Outer ring	29.5 (2.5)	28.4 (2.6)	29.2 (2.8)	0.15 (0.021)	0.73 (0.22)
4) INL					
Fovea	18.7 (4.6)	17.2 (3.1)	15.5 (2.8)	0.16 (0.82)	0.006 (0.019)
Inner ring	40.3 (2.7)	41.1 (3.6)	40.2 (2.7)	0.13 (0.33)	0.88 (0.50)
Outer ring	33.3 (2.4)	34.4 (2.4)	33.9 (2.5)	0.05 (0.78)	0.44 (0.47)
5) OPL					
Fovea	26.2 (5.3)	27.0 (4.3)	22.6 (3.4)	0.47 (0.76)	0.005 (0.002)
Inner ring	33.5 (3.8)	35.8 (4.6)	33.6 (3.7)	0.008 (0.06)	0.95 (0.92)
Outer ring	27.1 (1.7)	28.2 (2.2)	28.1 (2.3)	0.005 (0.005)	0.030 (0.014)
6) ONL					
Fovea	92.5 (10.3)	91.5 (9.7)	86.5 (5.8)	0.58 (0.93)	0.024 (0.11)
Inner ring	72.2 (8.6)	67.9 (9.5)	69.6 (5.0)	0.03 (0.12)	0.26 (0.37)
Outer ring	59.8 (6.5)	57.0 (7.4)	57.4 (4.6)	0.07 (0.043)	0.19 (0.09)
7) ORL (PR)					
Fovea	90.6 (4.1)	89.9 (4.4)	91.0 (4.9)	0.49 (0.30)	0.74 (0.64)
Inner ring	81.6 (2.3)	80.9 (2.2)	80.7 (2.4)	0.23 (0.11)	0.14 (0.06)
Outer ring	78.3 (2.1)	77.9 (2.5)	77.7 (2.0)	0.68 (0.25)	0.19 (0.11)
8) RPE					
Fovea	17.1 (1.8)	16.7 (1.7)	17.2 (2.0)	0.45 (0.43)	0.80 (0.82)
Inner ring	14.8 (1.5)	14.1 (1.2)	14.4 (1.2)	0.029 (0.28)	0.19 (0.68)
Outer ring	13.1 (1.2)	13.1 (1.2)	13.1 (1.1)	0.61 (0.25)	0.94 (0.70)

*OCT* optical coherence tomography, *SD* standard deviation, *GD3* Gaucher disease type 3, *PKU* phenylketonuria, *NFL* nerve fiber layer, *GCL* ganglion cell layer, *IPL* inner plexiform layer, *INL* inner nuclear layer, *OPL* outer plexiform layer, *ONL* outer nuclear layer, *ORL* outer retinal layer (being limited by the external limiting membrane and Bruch’s membrane, this layer corresponds to the photoreceptors), *RPE* retinal pigment epithelium, *adj. model* adjusted model

GCL correlated with current tyrosine serum concentration (outer ring: rho = 0.70, *p* = 0.0008 (< 0.001); inner ring: rho = 0.51, *p* = 0.025), as well as IPL did (inner ring: rho = 0.61, *p* = 0.006). ONL correlated inversely with current phenylalanine serum concentration (outer ring: rho =  − 0.59, *p* = 0.01; inner ring: − 0.63, *p* = 0.004). Early-treated PKU patients had rather thick GCL (inner ring: rho = 0.55, *p* = 0.014) and thick IPL (inner ring: rho = 0.54, *p* = 0.02) compared to late-treated patients.

#### Gaucher disease type 3

Thinning of total retinal thickness was found in all subfields (fovea, inner ring, and outer ring) compared to the controls, even after adjusting for sex, age, and spherical equivalent (Table 1). The retinal layers affected by thinning were NFL (outer ring significantly), GCL (inner ring significantly), and IPL (slightly), while OPL (outer ring) was thickened, and the remaining layers (INL, ONL, ORL, and RPE) showed no difference in thickness to the controls. Individual foveal layers were normal (when only considering the layers ONL outwards, because the layers NFL to OPL are very small and do not yield reliable OCT data in general). However, total retinal thinning was most evident in the fovea.

NFL outer ring correlated inversely with mSST (outer ring: rho =  − 0.49; *p* = 0.046; inner ring: rho =  − 0.39, *p* = 0.11), and OPL correlated inversely with horizontal peak velocity (OPL outer ring: rho =  − 0.57, *p* = 0.020; OPL inner ring: rho =  − 0.51, *p* = 0.040). In GD3, no correlations were found for foveal total retinal thickness, and GCL, and none with vertical peak velocity or phenotype severity (mild/intermediate/severe).

### Peripapillary RNFL

The global peripapillary RNFL did not differ significantly between the groups with 96 µm in PKU and GD3 and 96.7 µm in control eyes. Regarding the distinct peripapillary RNFL sectors, only the temporal-inferior sector was significantly thicker in PKU eyes compared to controls (*p* = 0.029), but the difference did not remain significant after adjusting for sex, age, and spherical equivalent (Table 2). The other sectors were normal in both diseases.

**Table 2:** Distribution of peripapillary retinal nerve fiber layer thickness from SD-OCT in phenylketonuria, Gaucher disease type 3, and controls. A linear mixed model was used for statistical analysis. In the adjusted model, age, sex, and spherical equivalent were included

Peripapillary RNFL Mean [µm]	Control eyes (*n* = 178)	PKU eyes (*n* = 30)	GD3 eyes (*n* = 24)	*p*-value PKU crude model [adj. model	*p*-value GD3 crude model [adj. model
Global (average)	96.7 ± 9.8	96.1 ± 9.5	96.0 ± 5.9	0.61 [0.63]	0.25 [0.19]
Temporal	69.9 ± 10.5	66.8 ± 10.2	71.1 ± 13.7	0.50 [0.14]	0.90 [0.36]
Temoral-superior	135.0 ± 18.0	134.8 ± 22.3	132.1 ± 20.0	0.71 [0.45]	0.88 [0.50]
Temporal-inferior	139.3 ± 19.3	148.5 ± 17.5	135.4 ± 21.0	0.029 [0.07]	0.33 [0.26]
Nasal	73.6 ± 15.6	71.1 ± 9.7	70.6 ± 11.1	0.39 [0.40]	0.47 [0.59]
Nasal-superior	106.3 ± 19.8	107.8 ± 19.1	109.3 ± 16.0	0.91 [0.85]	0.38 [0.58]
Nasal-inferior	105.8 ± 23.6	102.9 ± 17.1	107.8 ± 34.1	0.63 [0.29]	0.44 [0.98]

*RNFL* retinal nerve fiber layer, ±  = standard deviation, *GD3* Gaucher disease type 3, *PKU* phenylketonuria, *adj. model* adjusted model

## Discussion

This study is one of the first, reporting OCT measurements in phenylketonuria and Gaucher disease type 3, besides the publications from Serfozo et al. [16, 26] and Tantawy et al. [17]. Our main finding, GCC reduction, is in line with these two studies [16, 17, 26]. We additionally demonstrate that each of the individual components (NFL, GCL, and IPL) is reduced in thickness in phenylketonuria. A similar pattern of retinal thinning was evident in GD3. The retinal measurements found in both conditions resemble changes seen during aging: GCL and IPL both thin out, while OPL thickens with age [27].

### Macular thickness

Alterations of the ganglion cell complex in various neurodegenerative diseases overlap and tend to show similarities. We found GCC reduction both in PKU and in GD3.

#### Phenylketonuria

Our data support the findings of Serfozo et al., who reported total retinal thinning in the parafoveal and perifoveal region, sparing the fovea [26], in early-treated phenylketonuria, and reduced GCC thickness (average, superior, and inferior quadrants) [16]. We further demonstrated OPL thickening in the inner and outer ring. Inverse correlation of phenylalanine serum concentration with retinal measurements was, at most, inconsistently found [26]. We found tyrosine serum concentrations correlating with GCL and IPL, which might indirectly indicate low cerebral tyrosine and dopamine concentrations.

#### Gaucher disease

In line with previous investigations, we confirm significant retinal thinning in the NFL (outer ring), GCL (outer and inner ring), and IPL (inner ring) after adjusting for sex, age, and refraction, and thickening of the OPL in GD3. Our data thus attest reduced GCC as reported by Tantawy et al., who divided a cohort of GD patients aged 11 to 29 years into a group with parkinsonian features (*n* = 11) and a group without (*n* = 37), independently of the type of GD. Their results were that GCC thickness differed between young patients with parkinsonian features and those without parkinsonian features. However, between GD1 (*n* = 14) and GD3 (*n* = 34), GCC thickness did not differ significantly, although all GD patients together (93.1 (± 7.0) µm) differed from the controls (98.7 (± 9.6) µm) [17]. Similarly, thinning of the retinal GCC was associated with potential clinical markers of early neurodegeneration in GD1 (*n* = 11) or GBA mutation carriers (intermediate level of glucocerebrosidase activity) in a study conducted by McNeill et al. [20]. GD1 patients without neurodegenerative symptoms do not show retinal GCC thinning [19, 20], although subsections of the GCC thickness (e.g., of the outer macular GCC nasally and inferiorly) revealed significant thinning [19]. In GD1, no correlation was found between retinal measurements and disease severity [19]. In GD3, we detected a weak correlation between modified severity scoring tool, and retinal NFL, and no correlation between saccadic measurements and retinal layer measurements.

The pathways of retinal damage including that of retinal ganglion cells are not fully understood in both diseases. Changes in neurotransmitter metabolism are discussed to play a role. Retinal dopamine deficiency is discussed to play a role in primary retina degeneration and secondary loss of dopamine-regulated neurons [26]. Besides disturbances in neurotransmitter metabolism, other changes may lead to morphological changes of the retina in both disorders. In PKU, this might be a direct neurotoxic effect of phenylalanine [28]. In the lysosomal storage disorder GD, the reduced activity of β-glucocerebrosidase is associated with accumulation of α-synuclein, inhibition of apoptosis, and reduced mitochondrial function with associated oxidative stress [29, 30]. Clinically, vitreous fluid of GD3 patients may contain visible opacities as well as Gaucher cells, and high concentration of glucosylceramide [31, 32]. In addition, vascular abnormalities with tortuosity, and occlusion were discussed to induce retinal damage [19]. In the present cohort, only three patients presented increased tortuosity of retinal vessels bilaterally, one patient presented caliper changes of retinal vessels, and two patients showed peripapillary atrophy of the outer retina [22].

As follow-up data are missing, we cannot exclude that in GD3 metabolic imbalances during embryonic development already influence a normal retina development.

### Peripapillary RNFL

#### Phenylketonuria

While Serfozo et al. found that average pRNFL was reduced—without prove of reduction in the quadrants—in early-treated phenylketonuria [16], we did not detect reduction in pRNFL thickness. The values of our pRNFL data, however, are in line with those measured by Serfozo et al., namely 96.3 µm (± 9.9 µm) in early-treated phenylketonuria. The different outcome might be attributed to thicker pRNFL in their controls (101.9 ± 7.2 µm) compared to ours [16].

#### Gaucher disease

Peripapillary RNFL reduction as detected by Matos et al. in a GD3 patient is not confirmed by our data. In this case, the authors attributed their findings to either *glaucoma of normal pressure* or a *nervous degeneration* [33]*.* While no other studies have investigated pRNFL in GD3, Weill et. al reported abnormal pRNFL scans in one-third of GD1 patients with significant thinning of the average, superior, and inferior pRNFL [19]. This pattern of damage was supposed to match a magnocellular type, which is also characteristic in Alzheimer’s disease [34]. From our data, we cannot conclude a damage or a pattern of damage at the level of the optic disc primarily due to GD3.

### Limitations and perspectives

This study has several limitations. First, we did not have complete long-term blood biomarker data of the PKU group (mean phenylalanine serum concentrations over the past 5 or 10 years) for the analysis. We also could not provide data of parkinsonian motor signs (bradykinesia, rigidity, rest tremor) [17], or prodromal symptoms (hyposmia, cognitive impairment, hallucinations, depression, sleep disorders, and autonomic dysfunction) [20] in the GD3 group. Unfortunately, these data were not available in our cohort. Longitudinal data is still missing, which could elucidate whether progression occurs over time. This is even more important, as progression in neuro-ophthalmologic diseases is subject to high interindividual variation.

### Conclusion

This OCT study with PKU and GD3 patients confirmed that retinal thickness is reduced, to elucidate, at the level of the NFL, GCL, IPL, and ONL, while OPL is thickened in PKU patients. The same is held true for GD3 although with fewer significance. Individual follow-up examinations are required for evaluation and detection of a progression of retinal neurodegeneration. This is important, because current therapies for these conditions might interfere with progressive morphological changes of the retina.

## Data Availability

The data are included in the manuscript. Participants of the study did not agree for their individual raw dataset to be shared publicly, so supporting data is not available.
